# Implied Consent in Treating Psychiatric Emergencies

**DOI:** 10.3389/fpsyt.2020.00127

**Published:** 2020-03-13

**Authors:** Sarah H. D. Becker, Howard Forman

**Affiliations:** Department of Psychiatry and Behavioral Sciences, Albert Einstein College of Medicine, New York, NY, United States

**Keywords:** involuntary treatment, involuntary commitment, psychiatry and the law, patient autonomy, psychiatric policy

A significant percentage of psychiatric emergencies occur *outside* the psychiatric inpatient unit (1), such as in emergency rooms, outpatient clinics, and on medical floors. The existing literature on the legal, ethical, and practical considerations of compulsory treatment in psychiatric emergency is limited. The purpose of this article is to review the relevant legal and ethical background of treatment over objection in the United States of America, define the term “psychiatric emergency,” examine the legal and ethical bases for physicians to act in these situations, and suggest further areas for thought and research. It is our hope that the legal underpinnings of involuntary treatment in the United States, as well as consideration of the relevant ethical issues, will allow lawmakers and providers to create the ideal framework for involuntary treatment outside of inpatient units, wherever they live.

## Background

Prior to the landmark cases of the latter half of the twentieth century, society and the courts expected psychiatrists to treat patients against their will, even holding psychiatrists accountable for not doing so. For example, a New York court decision in 1968 awarded a patient $300,000 in damages because a hospital did not treat him against his will (2). The attorney general of Pennsylvania wrote that the legal purpose of hospitalization was treatment and therefore no consent was necessary prior to administration of electroconvulsive therapy in state hospitals (3). These decisions were consistent with the psychiatrist's role as state agent and his duty to execute the principles of *parens patriae* (lit. the father of his country), to protect vulnerable individuals, as well as *salus populi suprema lex esto* (lit. the welfare of the people shall be the supreme law), the state's police power to protect its citizens from others (4).

The pendulum from physician responsibility to patient rights would shift in the courtroom. *Rogers v. Okin* (5), in Massachusetts, raised the argument that overriding a patient's refusal of psychotropic medications violated his or her *constitutional* rights to free speech and mentation, privacy, due process, and freedom from cruel and unusual punishment (6). Furrow (7) in a 1982 article extended *Rogers v. Okin* to its “common law analog in tort, informed consent doctrine.” He writes that the right to refuse treatment stems from a person's autonomy and is most in line with “dignitary” torts—battery, invasion of privacy, and defamation. Battery is the non-consensual harmful or offensive touching between a perpetrator and victim, which in medicine would be the forcible treatment of an unwilling patient. A non-psychiatric example of this is *Pugsley v. Privette* (8), in which a woman was awarded $75,000 in damages for battery after she underwent a complicated bilateral oophorectomy for which she had consented to only on condition the procedure would be supervised by her general surgeon, who was not present for the surgery. Thomas and Moore (9) relate the case to treatment of an agitated patient without consent, while also raising concern for violation of the false imprisonment tort when using chemical or physical restraints or any means to hold a patient against his or her will. In *Barker v. Netcare Corp*., (10) the hospital was found liable for false imprisonment when it used physical and chemical restraints to hold Ms. Barker in the emergency room without commencing involuntary commitment proceedings.

The legal founding for patient autonomy in the healthcare system can be traced back to *Schoendorff v. Society of New York Hosp*. (11), when the eminent Justice Cordoza wrote: “Every human being of adult years and sound mind has a right to determine what shall be done with his own body.” The process by which we honor this patient autonomy is known as informed consent, wherein the physician explains the procedure, risks and benefits of either accepting or refusing, and then allows the patient to choose whether to proceed (12). The key part of the Schoendorff ruling is that the patient must be of a sound mind, for which the legal term is competence. The medical cousin of competence is capacity, the individual's ability to make an informed decision, which by definition is case specific (13). All persons are assumed to be both competent and have capacity until proven otherwise. As recently as 1953, Indiana state law stated, “Commitment to a hospital for the insane is equivalent to a prior adjudication of incompetency” (14). Over the course of the following decades, the courts would erode this presumed global incompetence for hospitalized psychiatric patients with the logical extension being the right to refuse treatment (15), as was seen in the NYS ruling Rivers v. Katz (16) where it was determined that not having capacity to refuse admission to an inpatient psychiatric unit does not necessarily render one incapacitated to refuse medication.

## Psychiatric Emergencies

Rivers v Katz (17) simultaneously prohibited psychiatric treatment over objection without due process in non-emergent situations for involuntarily committed patients while also establishing grounds for treatment of acute psychiatric emergencies when the patient poses an immediate or substantial threat of physical harm to himself or others. Given that the psychiatric emergency is the sole option for medication over objection in the extrajudicial setting (18), it is important to define what constitutes a psychiatric emergency. As will be seen, this is a matter of great debate.

With regard to medical emergencies, whether defined narrowly as threat of loss of life or limb or as broadly as a situation of “acute suffering” (19), there remains a physical condition which “is usually objectively demonstrable” (18). This objective observability is often lacking in psychiatric emergencies. In the original ruling of *Rogers v. Okin*, the district court judge narrowly defined a psychiatric emergency as “the substantial likelihood of physical harm” (20). This definition was rejected by the Court of Appeals (5) due to its near-impossible requirement for physicians to determine that the occurrence of harm was more probable than its absence. The definition was eventually broadened by the Massachusetts Supreme Judicial Court to include both “occurrence or serious threat of extreme violence, personal injury, or attempted suicide” as well as “necessity of preventing immediate, substantial and irreversible deterioration of a serious mental illness…in which even the smallest of delays would be intolerable” (21). The astute reader will recognize the former reason to be consistent with the physician's power to act as a state agent in police power emergencies and the latter an example of the physicians role as *parens patriae*. Indeed, the Rivers ruling by the New York Court of Appeals (16) parallels this dichotomy by defining psychiatric emergency both in terms of potential for imminent harm as well as potential for deterioration in mental health. In practice, despite evidence that prolonged untreated psychosis results in worse outcomes (22), *parens patriae* rationale is rarely invoked in treating psychiatric emergencies due to the difficulty proving “irreversible deterioration” with a finite delay in treatment (18).

## Emergent Treatment Outside of the Psychiatric Inpatient Unit

Both Rivers and Rogers are examples of case law applicable to the involuntarily hospitalized psychiatric patient. As noted in the introduction, a significant percentage of psychiatric emergencies occur outside the psychiatric hospital, whether that be in the emergency room, medical units, or other healthcare settings. In those settings, the relevant legal doctrine would likely be found in public health law. New York State Public Health Law (23) dictates that medical treatment may be rendered without consent if delay in treatment would “increase the risk to the person's life or health.” This definition provides a wide range for physicians operating under *parens patriae*, necessary for the day-to-day functioning of emergency departments. However, there is no mention of police power emergencies which are common occurrences in psychiatric emergency rooms nor is there mention of psychiatric patients being treated in medical emergency rooms. Though not referring specifically to psychiatric emergencies, the 2017 Report of the United Nations High Commissioner for Human Rights, titled “Mental Health and Human Rights,” asserts that “outside of institutions, the use of community treatment orders or mandatory outpatient treatment, even if enforced in the community, violates the right to liberty and security of the person as such measures impose treatment and the threat of detention if refused” (24).

Rice and Moore (25) suggest several justifications for the treatment of psychiatric patients in the emergency setting. Firstly, they posit that the very presentation of the patient to the emergency room implies consent for evaluation and care, just as it does for medical patients. Secondly, once a patient presents to the emergency room, a duty is established between physician and patient to provide the “standard care,” not doing so would be tantamount to negligence. While the former line of reasoning would only seem to apply to patients who present voluntarily to the emergency room, not those brought unwilling by family or emergency services, the latter would apply regardless of mode of presentation. While a competent patient can decline care, if the physician has reason to believe a patient lacks decisional capacity—as is often the case in psychiatric emergencies such as violent, psychotic, or suicidal behaviors—he is obligated to treat, or risks charges of negligence. Lastly, and perhaps most unusually, the authors extend the physician's “duty to warn” (26) to indicate the court sanctions and even requires providers to protect third-parties, especially if said third parties were under the hospital‘s care, such as other patients in the emergency room. This last reasoning would provide the legal justification for use of police powers outside of a psychiatric inpatient unit.

Several ethical considerations have been raised to promote forced medication of the agitated psychiatric patient in an emergency situation, including “goal of restoration of autonomy, reduced risk of harm, and treatment of the underlying condition” (12). Furthermore, the protection of staff in an ever-increasingly dangerous workplace (27) to ensure the continued staffing of emergency rooms for all patients is a legitimate public interest. From a practical perspective, articles considering the management of psychiatric patients in the emergency setting, invariably take for granted the physician's obligation to first ensure the safety of other patients and staff members (1).

## Conclusion and Future Directions

The responsibility of a physician to provide compulsory treatment in a psychiatric emergency is an important exception to the fundamental human right to make decisions about his or her own body and mind. The interplay of emergent need, presumed incompetence, implied consent, and state interest, along with the individual details of each case, are all important in making the correct ethical and legal decision in a given emergency.

While non-emergent treatment over objection decisions benefit from court oversight to ensure there is a compelling state interest to medicate, whether with regard to *parens patriae* or police power, the emergency situation does not allow for any delay in the decision making process. The Massachusetts' Appeals Court rejected the expectation that physicians could predict with reasonable accuracy the probability of imminent harm in a psychiatric emergency. However, legal standards or practice guidelines to determine the threshold for imminent danger would be welcome additions to the field for psychiatrist and patient alike. Furthermore, explicit legal guidelines specific to emergency psychiatric management of populations other than involuntarily admitted patients are sorely needed. The need is not limited to managing a patient's agitation or suicidality but extends to management of a patient in need of psychiatric admission who refuses medical clearance. These guidelines, along with physician education, would promote standardization of the psychiatric patient's experience in the emergency setting.

## Author Contributions

SB wrote almost the entire manuscript under guidance from HF. HF then edited the manuscript and returned it to SB for her approval of the changes.

### Conflict of Interest

The authors declare that the research was conducted in the absence of any commercial or financial relationships that could be construed as a potential conflict of interest.
